# An Easy-to-Use Tool to Predict SARS-CoV-2 Risk of Infection in Closed Settings: Validation with the Use of an Individual-Based Monte Carlo Simulation

**DOI:** 10.3390/microorganisms12122401

**Published:** 2024-11-22

**Authors:** Benedetta Santoro, Francesca Larese Filon, Edoardo Milotti

**Affiliations:** 1 Physics Department, University of Trieste, 34127 Trieste, Italy; benedetta.santoro@phd.units.it (B.S.); milotti@ts.infn.it (E.M.); 2 Occupational Medicine Department, University Hospital of Trieste, 34129 Trieste, Italy; 3 I. N. F. N.—Sezione di Trieste, 34149 Trieste, Italy

**Keywords:** SARS-CoV-2, closed setting, diffusion, model validation, Monte Carlo, risk evaluation, hospital setting

## Abstract

The dynamics of the SARS-CoV-2 pandemic showed that closed environments, such as hospitals and schools, are more likely to host infection clusters due to environmental variables like humidity, ventilation, and overcrowding. This study aimed to validate our local transmission model by reproducing the data on SARS-CoV-2 diffusion in a hospital ward. We implemented our model in a Monte Carlo procedure that simulates the contacts between patients and healthcare workers in Trieste’s geriatric ward and calculates the number of infected individuals. We found the median number of infected workers to be 38.98 (IQR = 7.75), while all patients were infected in most of the simulation runs. More infections occurred in rooms with lower volumes. Higher ventilation and mask-wearing contribute to reduced infections; in particular, we obtained a median value of 35.06 (IQR = 9.21) for the simulation in which we doubled room ventilation and 26.12 (IQR = 10.33) in the simulation run in which workers wore surgical masks. We managed to reproduce the data on infections in the ward; using a sensitivity analysis, we identified the parameters that had the greatest impact on the probability of transmission and the size of the outbreak.

## 1. Introduction

The virus that caused the COVID-19 pandemic, SARS-CoV-2, is a pathogen that likely spreads by means of droplets and diffusion [1,2] of airborne aerosol [3,4,5,6,7]. Owing to these paths of transmission, the SARS-CoV-2 virus, like other microorganisms, causes a higher risk of infection in closed environments where poor ventilation is more likely, especially during the winter, and in environments with overcrowding [8]. Studies on the transmission of SARS-CoV-2 have followed two main trends: epidemiology and local modeling. Many epidemiological models have been proposed. Starting from the basic SIR (Susceptible, Infected, Removed/Recovered) model [9], different research groups proposed models with more compartments to address the complexity of the COVID-19 pandemic, to take into account, for example, incubation (latency) time (i.e., time between exposure and onset of symptoms), different severity of the infection (asymptomatic, mild symptomatic, and severe symptomatic individuals), age differences, hospitalization, and vaccination [10,11,12]. Data-driven algorithms have also been considered [13]. Because closed environments are recognized as areas at risk owing to the higher probability of transmission [14], different aerosol diffusion models were proposed to evaluate the infection probability. The starting point for many of these studies was the Wells–Riley model [15] of diffusion, which was modified according to the specificity of the new pathogens; otherwise, other groups used other simulation techniques that involved the use of computational fluid dynamics [16]. Given the great variability in many problem parameters and the practical impossibility of precisely defining the environment of hospital rooms, it is often not feasible to perform fluid dynamics calculations. Therefore, we adopt a different, user-friendly approach [17] based on that proposed by Riley [15], where we adopt an average description of the hospital rooms and use a Monte Carlo simulation to validate its predictions with data from an infection cluster that occurred at the University Hospital of Trieste, in which healthcare workers were involved and followed up for infection and prevention controls. This study aims to demonstrate that our model is reliable for estimating the number of infections through the results of a simple numerical simulation. We also suggest using this tool to predict the risk of infection in closed spaces for preventive purposes because of its straightforward adaptation to different airborne pathogens.

## 2. Materials and Methods

### 2.1. Mathematical Model

We implemented a model that is a modified version of the Wells–Riley model. Because data provided by the hospital are generally insufficient to simulate the airflow in the ward through fluid dynamics and are very likely to remain so in all possible future occurrences of similar infections, we opted to simulate an average scenario and assume that the air volume in the region of interest is well mixed, with a uniform distribution of the pathogen concentration. The probability of at least one infection follows from Poisson statistics and is given by [18]:

(1) $$1\;-\;\exp(-\bar{I}),$$

where $\bar{I}$ is the number of pathogenic particles inhaled by susceptible individuals and is given by [18]:

(2) $$\bar{I}\;=\;\left(1-\alpha \right)\left(1-\beta \right)\;I\;\frac{rp}{\sum_{i}\lambda_{i}V}t.$$

In this equation:

- α and β are the outward and inward mask filtration efficiency, respectively;
- *I* is the number of infectious individuals, *r* is the quanta emission rate, *p* is the pulmonary ventilation rate, *V* is the room volume, and *t* is the exposure time;
- $\lambda_{i}$ are decay constants accounting for removing mechanisms; in particular, we considered ventilation, relative humidity, solar illumination, and droplet deposition.

The specific values of these parameters are detailed in the Section 3. These values are derived from the literature and experimental data specific to the infection type and hospital conditions.

### 2.2. Algorithm of the Monte Carlo Simulation

#### 2.2.1. Initial Conditions

The simulation starts by setting the initial scenario:

- Number of patients and healthcare workers. Let $N_{p}$ represent the number of patients hospitalized in the ward and $N_{w}$ the number of infected workers. Patients are distributed across $N_{r}$ rooms.
- Room assignment. Each patient $P_{j}$ (*j* = 1,…$N_{p}$) is randomly assigned to room $R_{j}$, chosen from the list of occupied rooms, with the corresponding volume $V_{j}$.
- Controlled environment. To model real hospital conditions, ventilation, relative humidity, and illumination are assumed to be controlled uniformly across the ward.

#### 2.2.2. Choice of Patient Zero

At the start of the simulation, one patient is randomly selected as patient zero, $P_{0}$, and is set as infectious with a latency time $\tau_{0}=0\;\mathrm{days}$, meaning that they begin spreading the disease immediately. The patient’s infectious status, which is represented by a binary value ι, is set to true ($\iota_{0}$ = 1). For other patients and workers, the latency time $\tau_{i}$ is randomly sampled from a specific distribution $f(\tau_{i})$ as explained in Section 2.3. Their infectious status is set to false ($\iota_{i}$ = 0).

#### 2.2.3. Computation of Infection Probability

Each day, $T_{day}$, workers and patients interact within the ward. Each day, a group of workers $A_{w}\;\left(A_{w}\leq N_{w}\right)$ is admitted to the ward and each of them visits some rooms singularly. If worker $W_{j}$ ($j=1,\ldots ,A_{w}$) encounters the infectious patient $P_{k}$ ($k=1,\ldots ,N_{p}$), the probability of infection is computed according to a Bernoulli distribution:

(3) $$\begin{array}{c} P_{infection,W}\;∼\;\mathrm{Bernoulli}\left(k\right), \end{array}$$

where *k* is computed according to Equation (1). If $W_{j}$ is infected (i.e., $P_{infection,W}$ = 1), his or her infection status is updated, but he or she becomes infectious at the end of their latency period $\tau_{j}$. The day on which they become infected is given by:

(4) $$\begin{array}{c} T_{infectious,W}\;=\;T_{day}+\tau_{j}. \end{array}$$

Otherwise, if an infectious worker $W_{j}$ enters a room with at least one susceptible individual, the probability of infection is calculated similarly.

Moreover, for patients sharing the same room $R_{j}$, if one of the occupants is infectious, the infection probability is computed using the same principles. Multiple infections are not allowed.

#### 2.2.4. Time Evolution of the Outbreak

The process is repeated each day $T_{\mathrm{day}}$ until the end of the epidemic.

#### 2.2.5. Iterations of the Simulation Runs

To achieve robust statistical results, the entire simulation process is repeated over 100,000 independent Monte Carlo simulation runs. Each run provides an estimate of infection outcome, allowing us to study the variability in outbreak spread under different initial conditions. By averaging over a large number of runs, we obtain the expected magnitude of the outbreak.

For a detailed explanation of the code structure and implementation, please refer to the Supplementary Materials.

### 2.3. Distribution of the Latency Periods

Since the evolution of the infectiousness of the occupants with time remains unknown, we decided to run the simulation twice by using two different sets of initial conditions, corresponding to two simple distributions of latency times for both patients and healthcare workers, to understand the dependence of the results on the knowledge of the distribution of the incubation period. In the first case, we assumed a uniform distribution, whose probability density function (PDF) is given by:

(5) $$\begin{array}{cc} PDF_{uniform}\left(x\right) & =\left\{\begin{array}{cc} \frac{1}{b-a} & \mathrm{if}\;x\in (a,b)\\ 0 & \mathrm{if}\;x\notin (a,b) \end{array}\right.; \end{array}$$

In the second case, we used the gamma distribution which has the following PDF:

(6) $$\begin{array}{cc} PDF_{gamma}\left(x,\delta ,\theta \right) & =\left\{\begin{array}{c} \frac{1}{\Gamma \left(\delta \right)\theta^{\delta }}x^{\delta -1}e^{-x/\theta } \end{array}\right.; \end{array}$$

where δ and θ are the shape and scale parameters, respectively, and Γ(δ) is a gamma function defined as:

(7) $$\Gamma \left(\delta \right)\;=\;\int_{0}^{\infty }t^{\delta -1}e^{-t}dt,$$

where the δ parameter is loosely associated with the number of accidental events, while θ is the average time interval between events.

### 2.4. Distribution of Infected Workers

The resulting distribution of infected workers turned out to be asymmetrical, as discussed in the following sections. Therefore, we described the data trend using a phenomenological asymmetrical (skewed) Gaussian distribution:

(8) $$PDF_{skewed-Gaussian}\left(x\right)=\frac{2}{\omega }\phi \left(\frac{x-\xi }{\omega }\right)\Phi \left[K\left(\frac{x-\xi }{\omega }\right)\right],$$

where ξ is the location of the peak, ω is the scale parameter, *k* is the shape parameter, ϕ(x) and Φ(x) are the standard normal probability density function (i.e., a Gaussian distribution with null mean and standard deviation equal to 1) and cumulative distribution function, which are defined as follows:

(9) $$\left\{\begin{array}{c} \phi \left(x\right)\;=\;\frac{1}{\sqrt{2\pi }}e^{-\frac{x^{2}}{2}}\\ \Phi \left(x\right)\;=\;\int_{-\infty }^{x}\phi \left(t\right)dt\;=\;\frac{1}{\sqrt{2\pi }}\int_{-\infty }^{x}e^{-\frac{t^{2}}{2}}dt \end{array}\right..$$

**Figure 1:**
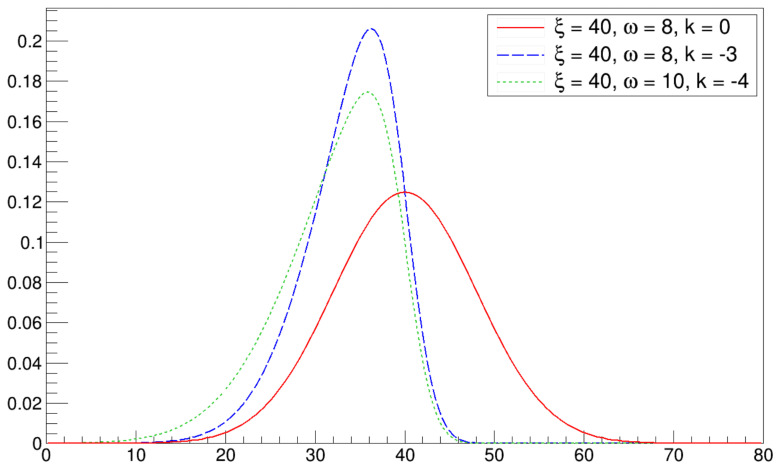
Four examples of the curve described by Equation (8), with different values of the parameters.

Four examples of the curve described by Equation (8) with different parameter values are shown in Figure 1. The red solid line represents the standard normal distribution, while the blue dashed line and green dotted curve represent two examples of skewed Gaussian distributions. The negative value of the *k* parameter for both distributions means that the curves are left-skewed, that is, values smaller than the mean are more probable than those larger than the mean.

### 2.5. Effect of Containment Strategies

We repeated the simulation by varying two parameters of the model, room ventilation and the use of personal protection equipment, to quantify how these strategies can be effective in reducing the spread of the disease.

## 3. Results

### 3.1. The Simulation Scenario for the COVID-19 Clusters in the University Hospital of Trieste

**Figure 2:**
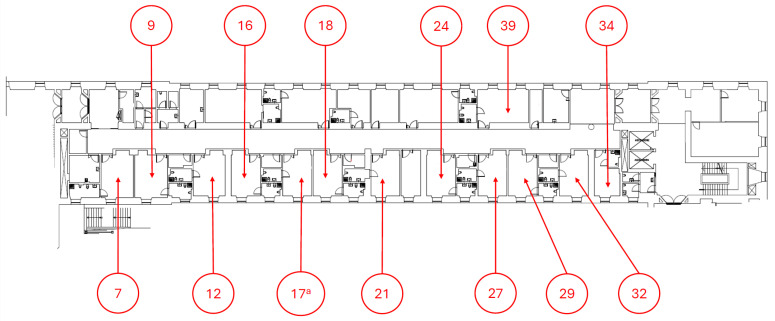
Plan of the Geriatric Department of the University Hospital of Trieste; numbers indicate patients’ rooms, as reported in Table 1.

The simulation scenario was set in the geriatric ward of the University Hospital of Trieste, where $N_{p}=26$ patients were arranged in $N_{r}=13$ rooms with two beds each, and where $N_{w}$ = 54 healthcare workers were allowed during the days of the outbreak. None of the occupants wore masks, which means that the factors α and β in Equation (2) are equal to 0. The plan of the ward is shown in Figure 2; red arrows indicate the patients’ rooms, the volumes $V_{i}$ of the rooms are reported in Table 1, and the mean ventilation is 2.5 air exchanges per hour, with an indoor relative humidity of 40% (see [18] for the corresponding decay constant). We assume the worst-case scenario in which infectious individuals have the highest quanta emission rate and the highest infectiousness [18]. From data provided by the Protection and Prevention Service of the University Hospital of Trieste, the average permanence time of a healthcare worker in a room is 0.30 h, and the number of admitted personnel ($A_{w}$) is fourteen workers per day. The values of the parameters in Equation (2) are collected in Table 2.

### 3.2. Distribution of Latency Periods

**Figure 3:**
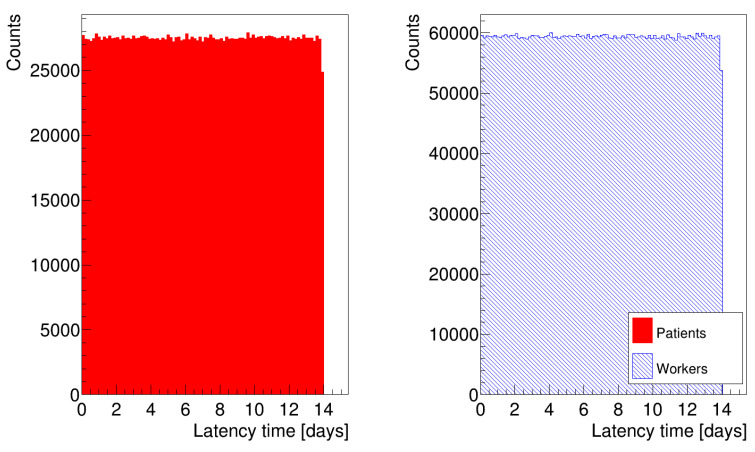
Distribution of latency times for patients and healthcare workers, generated according to Equation (5).

**Figure 4:**
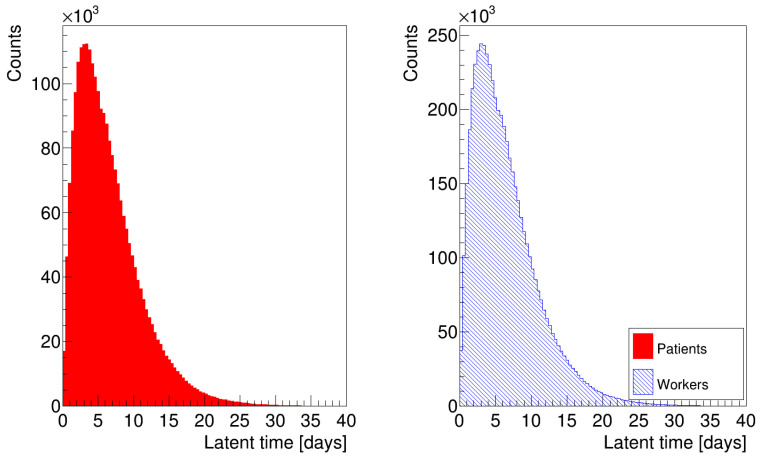
Distribution of latency times for patients and healthcare workers, generated according to Equation (6).

The histograms in Figure 3 show the distribution of latency times for patients and healthcare workers, where we generated random numbers according to Equation (5). We removed all data referring to patient zero, who has a latency time $\tau_{0}$ equal to 0, to avoid peaks at t = 0, which would have biased the distributions. The histograms in Figure 4 are analogous to the previous ones, but we generated the latency times for the occupants according to Equation (6). We assigned a different latency time to each individual to account for differences in susceptibility and immune response. We based the choice of the parameters in Equations (5) and (6) on data obtained from the hospital about the dates of encounters between infectious individuals and the onset of symptoms. In particular, we chose a=0 and b=14 days as extreme values of the uniform distribution, and δ=2 and θ=3.3 for the gamma distribution. In the simulation, we also considered that individuals might spread the disease one day before the end of the latency period [23], which means that the time between infection and infectiousness (latent time) was shorter than the latency time.

### 3.3. Distribution of Infected Individuals

**Figure 5:**
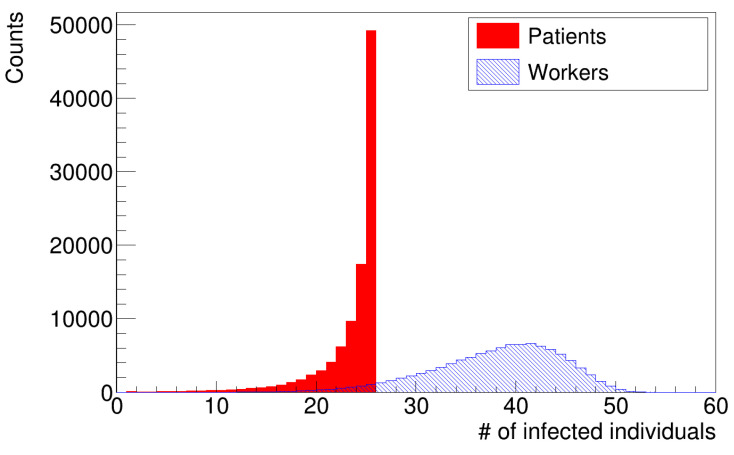
Distribution of the number of infections (uniform distribution of latency times).

**Figure 6:**
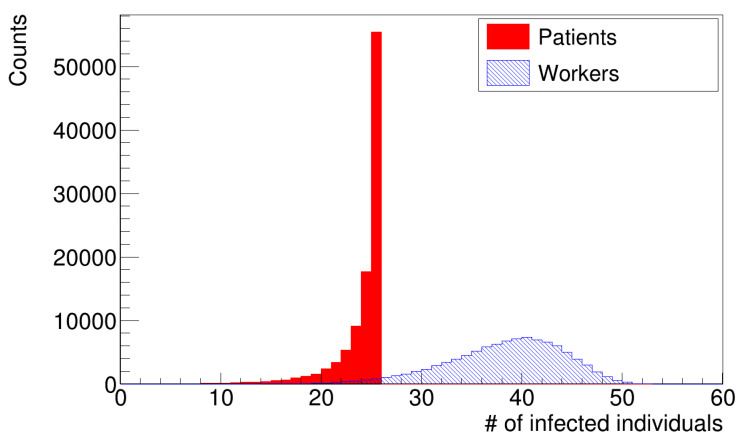
Distribution of the number of infections (gamma distribution of latency times).

Figure 5 and Figure 6 show histograms of the number of infections for the two simulation scenarios with different distributions of latency times. The distributions of infected patients (histograms in red) were truncated to the value of twenty-five patients, which is the number of susceptible patients at the beginning of the simulation. The peak of the distribution was in agreement with data provided by the hospital.

**Figure 7:**
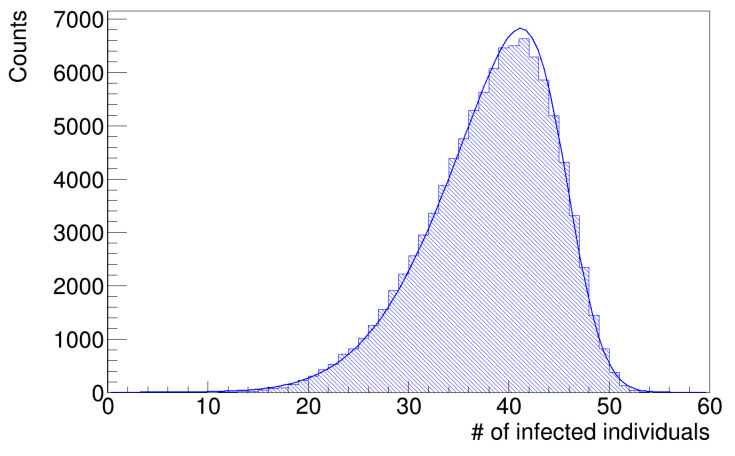
Number of infected workers (uniform distribution of latency times).

**Figure 8:**
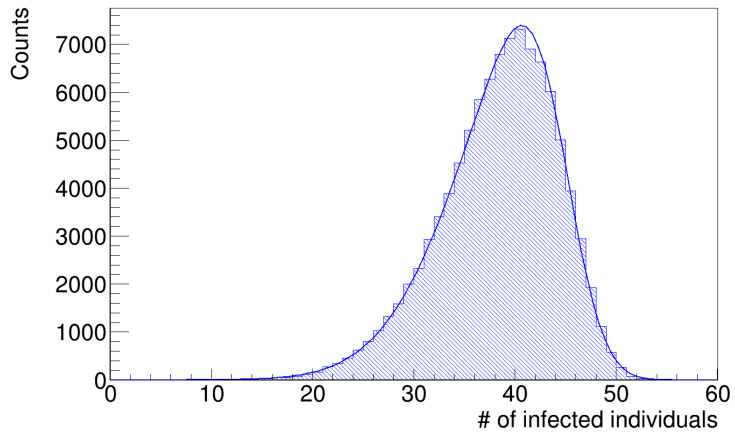
Number of infected workers (gamma distribution of latency times).

As anticipated in the Section 2, the histograms of infected workers are asymmetrical. Figure 7 and Figure 8 show the histograms of the number of infected workers with the trend line given by Equation (8), whose parameters are reported in Table 3. These distributions are left-skewed; indeed, the *k* parameter in Table 3 is negative, which means there is a higher fraction of simulation runs in which the number of infected workers is less than the mean value. Since the distribution is skewed, the mean value is not a good estimator of the most probable value; therefore, we computed the median values of the histograms in Figure 7 and Figure 8 as better estimators and the interquartile ranges (IQR), defined as the difference between the 75th and 25th percentiles of the data, to determine their statistical dispersion. The parameters are listed in Table 4.

These median values are in good agreement with the actual value of 39 infected workers, with fractional errors of 0.13% and 0.05%, respectively. These results show that the simulation with the gamma distribution of latency times better reproduces the original data, even if the differences are small (see Section 4 below).

**Figure 9:**
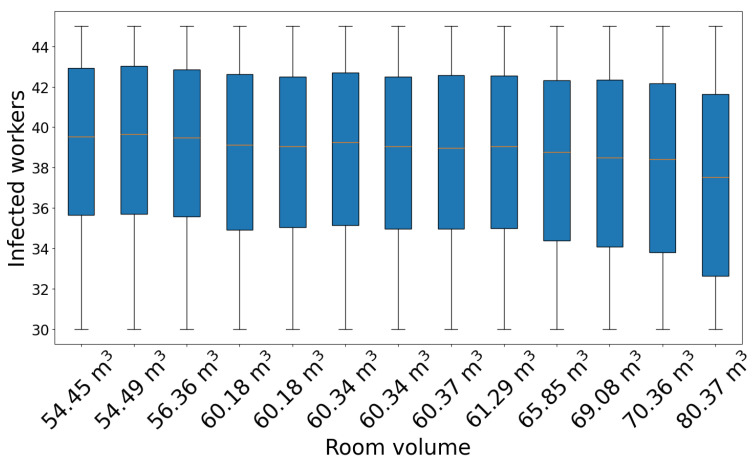
Box plots of the number of infected workers vs. room volume. The median value is indicated by the yellow horizontal line (gamma distribution of latency times).

We determined that a higher fraction of infections for healthcare workers occurred during the first days of the outbreak due to encounters with patient zero and the other occupant of the room, who quickly became infected. For this reason, we studied the dependence of the final size of the infection cluster on the volume of patient zero’s room. Because ventilation, relative humidity, illumination, and occupancy were the same for every room in the ward, room volume was the only parameter that changed in the formula of the infection probability. The results are presented in Table 5, where we indicate the room number, volume, median value of the distribution of infected workers, and the IQR. The same data are shown in the box plot in Figure 9 (we have truncated the whiskers for better data visualization). From this graph, it is possible to observe a smooth decrease in the cluster size with increasing volume.

**Figure 10:**
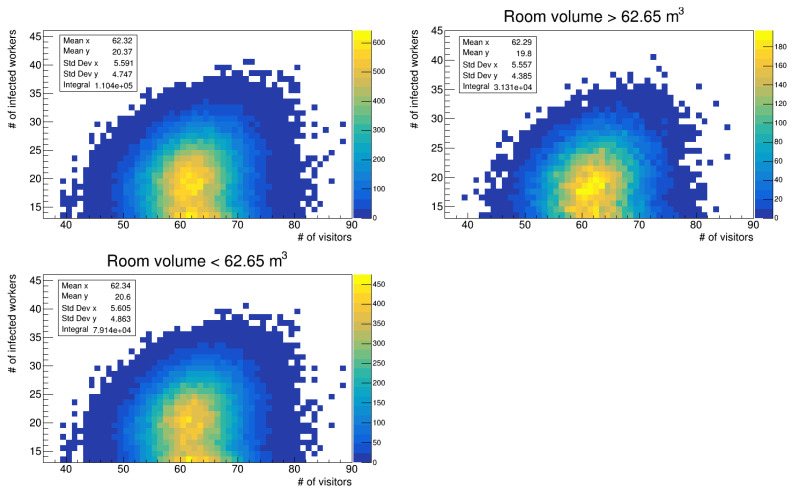
Number of infected workers vs. number of visits (uniform distribution of latency times). **Top left** panel: all room volumes; **top right** panel: volume >62.65 m^3^; **bottom left** panel: volume <62.65 m^3^.

**Figure 11:**
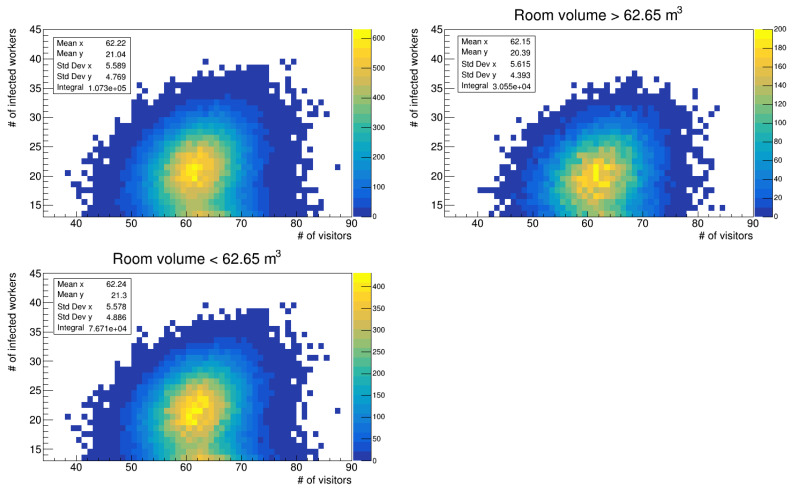
Number of infected workers vs. number of visits (gamma distribution of latency times). **Top left** panel: all room volumes; **top right** panel: volume >62.65 m^3^; **bottom left** panel: volume <62.65 m^3^.

We also studied the distribution of infected workers with respect to the number of visits, particularly in the region with more than 15 infections (see the top left panels in Figure 10 and Figure 11). We further subdivided the data into two groups: one for data referring to rooms with a volume higher than the mean of 62.65 m^3^ (see top right panels in the figures) and one for those with a volume lower than the mean (see bottom left panels). While the mean number of visits remains almost constant between the whole data distribution and the cuts, there is a difference in the mean values of infections that occurred in the room, that is, the mean value on the y-axis. In particular, for data referring to the simulation run with a uniform distribution of latency periods, the mean value of infections in smaller rooms is higher than that of the total distribution, with a difference of 1.1%, while the mean value for larger volumes is lower with a fractional shift of 2.8%. For the run with the gamma distribution, the trend is the same; however, we computed a fractional difference of 1.2% and 3.1%. The differences are small, but given that the infection probability depends on exp(-c/volume) (c represents all other parameters in the calculation that do not change), we did not expect larger differences due to room volume. Given the integral of the data reported in the aforementioned figures, the fraction of workers who get infected in larger rooms is 28.3% (28.5%) of the total distribution, almost two and half times less than those infected in smaller rooms; thus, the volume of the environments is an important factor to be considered to reduce infection probability.

### 3.4. Effect of Ventilation and Mask Wearing

**Figure 12:**
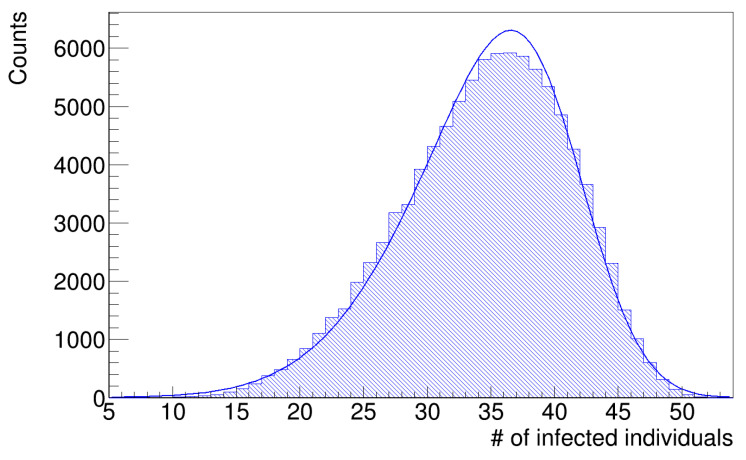
Number of infected workers (gamma distribution of latency times). In the simulation, we doubled the air exchange rate compared to the simulations that produced the data in Figure 8.

**Figure 13:**
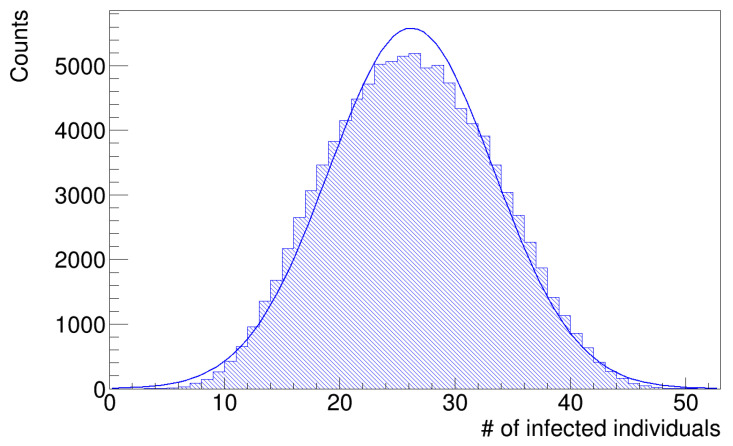
Same as Figure 8, but with healthcare workers wearing surgical masks.

**Figure 14:**
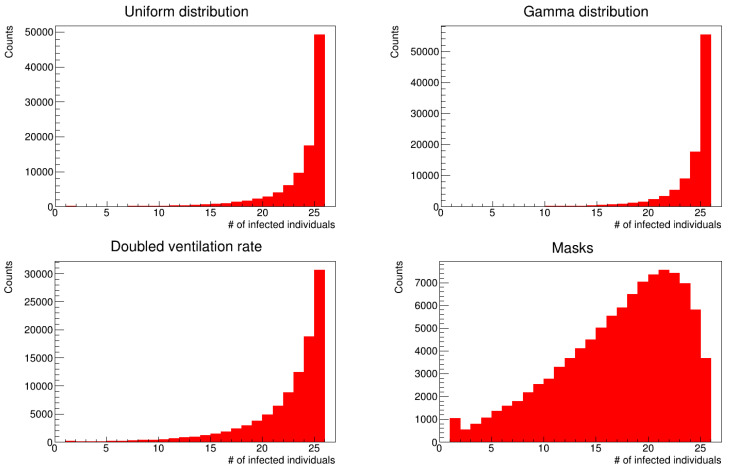
Empirical probability distributions (not normalized) of the number of infected patients in four different settings. **Top left** panel: uniform distribution; **top right** panel: gamma distribution; **bottom left** panel: gamma distribution and doubled rate of air exchanges; **bottom right** panel: gamma distribution and healthcare workers wearing surgical masks.

**Figure 15:**
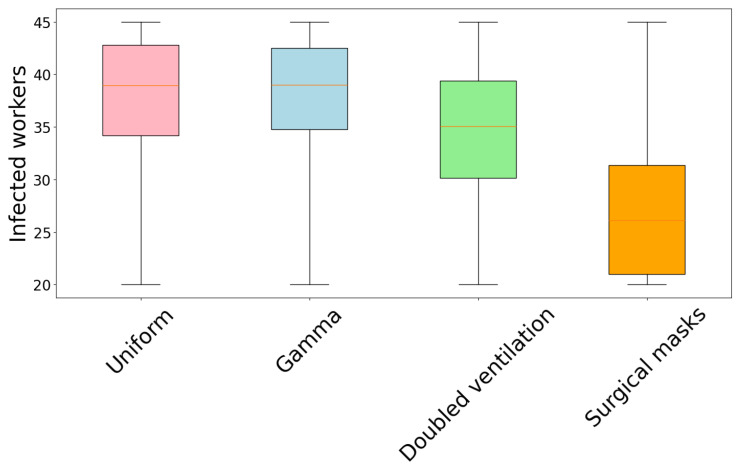
Box plot summarizing the histograms of the infected workers of the four numerical simulations.

To show the effect of the environmental parameters, we repeated the simulation by doubling the ventilation of each room, thus setting it to five air exchanges per hour while keeping the other parameters constant. The resulting histogram of the number of infected workers is shown in Figure 12, with mean and median values of 34.01 ± 6.59 and 35.06 (IQR = 9.21), while the parameters of the skewed Gaussian distribution are reported in Table 6. Finally, we modified the simulation code and allowed healthcare workers to wear surgical masks during daily visits. The results are shown in Figure 13, where the mean and median values of this histogram are 25.70 ± 7.14 and 26.12 (IQR = 10.33), respectively. Because the value of the *K* parameter from Equation (8) was compatible with zero, we used a standard Gaussian distribution with mean μ and standard deviation ω; see Table 7. The small difference between the mean and median values indicates that the data are approaching a Gaussian distribution. In the simulation, the outward and inward protection effectiveness are different [18,24], with the former being higher than the latter, which means that masks protect patients from being infected by workers rather than prevent workers from becoming infected. In particular, we assumed α=0.53 and β=0.49 [24] Figure 14 shows the resulting distributions of infected patients for the four simulations. The distribution corresponding to the simulation with surgical masks (bottom right panel) does not have a peak value at 25 infected patients, but it reaches a peak at 23 and then decreases, which shows the degree of the preventive efficacy of masks. For simulations with doubled ventilation and surgical masks, we used the gamma distribution for the latency periods. Finally, we summarize the results of the four numerical simulations in the box plot in Figure 15.

### 3.5. Evaluation of the Statistical Uncertainty on the Median Values

**Figure 16:**
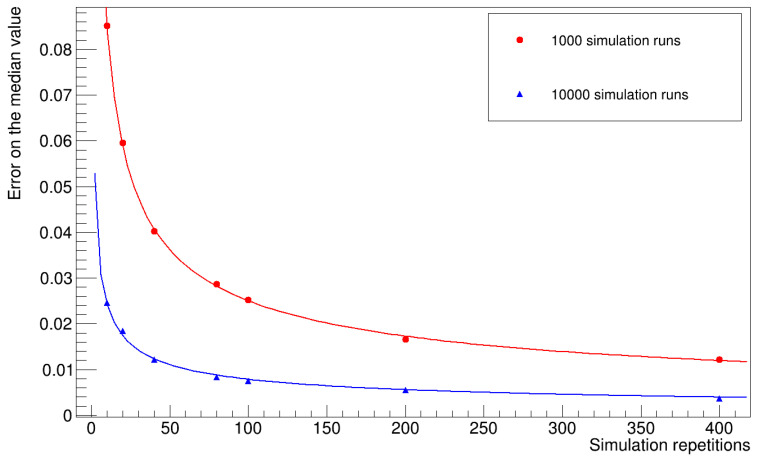
The plot shows the statistical uncertainty of the median of the number of infected workers (uniform distribution of latency times).

**Figure 17:**
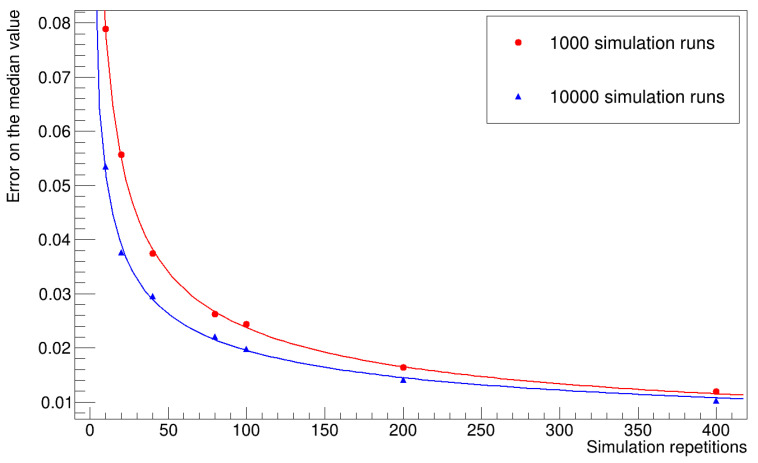
The plot shows the statistical uncertainty of the median number of infected workers (gamma distribution of latency times).

To evaluate the statistical uncertainty associated with the median values, we repeated the entire simulation several times, computed the median value of the histogram of the number of infected workers for each repetition, and calculated the standard deviation of the mean value of the sample we obtained. Figure 16 and Figure 17 summarize the results obtained for different numbers of repetitions and simulation runs. The uncertainty decreases with increasing sample size, and the trend lines in the graph approximate one over the square root of the sample size, and with the number of simulation runs for each repetition, then stabilizes at a certain value. We expect to improve this evaluation by increasing the number of runs.

### 3.6. Sensitivity Analysis

We have already discussed the impact of ventilation and mask-wearing in reducing the size of the outbreak.

In this section, we present the results of a sensitivity analysis we conducted to quantify the impact of the choice of the simulation parameters. In particular, we focus on:

- The parameters of the two distributions of the latency times, i.e., *b*, δ and θ in Equations (5) and (6);
- Time difference between the start of the infectiousness window and onset of symptoms.

For this analysis, we choose the OAT (one-factor-at-a-time) method [25]. If the output of the simulation, in our case the value of infected workers, is written as a function of all the parameters $O(q_{1},\ldots q_{n})$, we repeat the simulation by varying each of the parameters above by ±1% while keeping the other n−1 parameters constant.

For each parameter *q*, we compute the relative variation, which is defined as:

(10) $$C\;=\;100\times \frac{|O\left(q_{+}\right)-O\left(q_{-}\right)|}{O(q)},$$

where $O(q_{+})$ and $O(q_{-})$ are the mean number of infected workers resulting from the simulation run in which we change *q* by ±1%, respectively, and O(q) is the mean number without parameter modification.

The results of the sensitivity analysis are reported in Table 8.

The parameters with the largest relative variation are *b*, δ, and θ, which have a greater impact on the infectivity window and therefore modify the simulation result; however, these differences can be considered negligible in terms of risk prediction as we have already seen that overall, the gamma distribution provides a reasonable approximation and results that are nearly indistinguishable from those provided by a uniform distribution of the latency times. Notably, we find that the results show a very low sensitivity on the “time difference” parameter.

## 4. Discussion

In this study, we validated our user-friendly tool for biological risk assessment in closed workplaces for the transmission of SARS-CoV-2 [18] by implementing it in a numerical simulation to reproduce data of the infection cluster that occurred in March 2020 in the geriatric ward of the University Hospital of Trieste.

We implemented the α and β parameters in the original model by Riley [15], which account for the shielding effect of the masks. In contrast to other works in the literature [19], we used them as a multiplying factor rather than introducing them as an additional decay constant $\lambda_{i}$. Indeed, masks mechanically shield the particles and reduce the number of pathogenic particles inhaled by susceptible individuals. Moreover, we considered relative humidity, solar illumination, and deposition as reducing factors, along with ventilation.

### 4.1. Dependence of the Results on Initial Conditions

We repeated the simulation with two distributions of latency time, considering the differences with the latent period, and obtained marginally better results for the simulation with the gamma distribution, with a smaller fractional difference from the actual data. Because the difference between the results obtained with the two distributions is small, the choice of the distribution of latency times is not a determining factor for the final results of the simulation.

### 4.2. Role of Environmental Variables in the Infection Dynamics

We analyzed the parameters that had the greatest impact on the size of the infection cluster. First, we determined how room volume could contribute to reducing the number of infections by comparing the number of infected workers in rooms with smaller volumes with those in larger rooms. We also recognized a positive trend in the size of the cluster with decreasing room volume, which hosts patient zero.

Many models in the literature [26] suggest that mitigation measures such as controlled ventilation through monitoring systems, continued mask-wearing, and strategic control of room occupancy are effective against the spread of the disease. The important role of ventilation has already been demonstrated in various models [26,27,28], in which the air change per hour rate and the fraction of outside air emitted in the room volume are crucial in reducing viral transmission. In the case of mechanical ventilation, the purification system, along with the airflow rate, can dramatically reduce exposure, particularly if high-efficiency particulate air (HEPA) filters are used [29]. In agreement with these results, our simulations showed that higher ventilation could reduce the cluster size.

### 4.3. Role of Mask-Wearing

Moreover, it was demonstrated that mask-wearing also has a protective effect on patients and reduces the probability of transmission from infected workers wearing them [29,30], which agrees with the results obtained in the simulation run in which we introduced masks; see Figure 14.

Thus, this study shows that their use must be encouraged, especially in wards with more susceptible individuals, such as geriatrics. In Italy, the use of personal protection equipment was already mandatory for workers in some wards at risk, like the infectious disease ward, before the pandemic and was subsequently extended to all departments during the peaks of the epidemics, but now we are returning to the conditions pre-dating COVID-19.

### 4.4. Conclusions and Future Work

We stress that a modified version of the Wells–Riley model of transmission, such as the one we presented, can be adapted to other pathogens like influenza, as reported in other works in dental settings [31] and that our results corroborate previous findings on the efficacy of ventilation and mask-wearing in reducing diffusion of other similar pathogens and the spreading of nosocomial events [32]. For preventive purposes, the determination of the optimal forced ventilation of spaces can be useful also in the design of new buildings and reorganizations of existing ones to address the problem of occupants’ safety from biological hazards and can be combined with energy efficiency studies. A similar approach was proposed by Guo [33], who combined the model of Wells–Riley with the spatial flow impact factor, intending to control the infection risk in a built environment and make the best use of the space and resources to curb the spread of infectious disease, even though these authors did not consider the effects of mask-wearing. Moreover, the need to estimate the absolute ventilation rate is crucial also in rooms where occupancy levels vary [34] to limit indoor transmission.

Jones [35] proposed a model that permitted obtaining a Relative Exposure Index as a function of space volume, viral emission rate, exposure time, occupant respiratory activity, and room ventilation. However, his model did not consider the probability of infection and the mitigation effect of mask-wearing. As mentioned in the introduction, other models involving computation fluid dynamics were proposed, such as that of Vuorinen [36], who used a pure Monte Carlo method to compute the exposure time needed to become infected in different public indoor environments.

We stress that our model returns numerical values of the infection probability and diffusion of the disease among occupants in closed spaces that are consistent with epidemiological data. For that reason, our approach permits us to perform a reliable risk assessment evaluation in closed spaces and hints at how to improve work conditions.

In this context, our tool should not be assimilated to other risk assessment frameworks [37], which are mostly used in clinical practice and define risk through scoring systems that might mislead decisions and are difficult to apply to single-case scenarios.

The strength of our method is the ease of use of the available software tool: COVID-19-Evaluation Tool, version 3 [17] used for risk assessment in workplaces as demonstrated by its validation using data of infection clusters that occurred in hospitals that we presented in this work. A comprehensive set of factors was considered in the model, such as different room volumes and ventilation and especially the crucial role of masks in improving safety. Moreover, the sensitivity analysis we presented in Section 3.6 shows how our model is robust with respect to changes in parameter values and can reproduce the observed values of the infected individuals with reduced uncertainty. Therefore, the model can be used to predict the risk of nosocomial events and is highly reproducible. As a limitation of our work and a possible topic of investigation for future work, we mention that the model does not consider super-spreading events in which tiny droplets released by infected people through coughing and sneezing propagate very quickly and reach large distances from the spreader [38], and the model does not take into account differences in the size of the droplets and their diffusion dynamics. However, we considered the definition of emission quanta according to the literature [15] and aimed to model an average scenario, even though the probability of infection might be overestimated. For a non-hospital environment, where the conditions of the rooms are more stable, it would be possible to perform a fluid dynamic simulation to better describe the airflow and diffusion of the viral quanta. Furthermore, we suggest a model with parameters that can be easily managed by professionals in healthcare and have an immediate confirmation of the clinical outcome.

Indeed, the tool [18] was distributed to members of the Prevention and Protection Service for their evaluations of the infection probability and provides suggestions on how to reduce risk in single rooms. Overall, this user-friendly tool can predict the outbreak of SARS-CoV-2 infections in a closed setting and can be used for risk assessment. Furthermore, it is possible to reduce risk by adopting the suggested preventive measures (ventilation, use of personal protective equipment, time of exposure, number of workers exposed, etc.).

Lastly, this work can be extended to other environments, like school classrooms or offices, to test its applicability. Given the escalating number of COVID-19 cases, we are confident that our work will help to raise attention to the use of passive containment strategies, which can be easily implemented, and help in their evaluations to prevent diffusion in environments at risk. 

## Figures and Tables

**Table 1:** Room volumes and occupations of the Geriatric Department of the University Hospital of Trieste supplied by the Protection and Prevention Service.

	Volume [m^3^]	Beds
Room 7	70.36	2
Room 9	69.08	2
Room 12	65.85	2
Room 16	61.29	2
Room 17a	56.36	2
Room 18	60.34	2
Room 21	60.18	2
Room 24	60.18	2
Room 27	60.37	2
Room 29	60.34	2
Room 32	54.45	2
Room 34	54.49	2
Room 39	80.37	2

**Table 2:** Parameter values used in Equation (2).

Parameter	Symbol	Value	Source
Outward mask efficiency	α	0.0	No mask
Inward mask efficiency	β	0.0	No mask
Quanta emission rate	*r*	2856.0 quanta/h	[19]
Pulmonary ventilation rate	*p*	0.48 m^3^/h	[20]
Exposure time	*t*	0.30 h	Hospital staff
Decay due to ventilation	$\lambda_{vent}$	2.5 h^−1^	Hospital staff
Decay due to RH	$\lambda_{RH}$	0.158 h^−1^	[19] for RH = 40%
Decay due to solar illumination	$\lambda_{UV}$	7.26 h^−1^	mean value from [21]
Decay due to droplets deposition	$\lambda_{dep}$	0.24 h^−1^	mean value from [22]

**Table 3:** Fit results on histograms of infected workers for the geriatric ward scenario. We report an *N* parameter due to the normalization of the data.

	Normal. [*N*]	Scale [ω]	Location [ξ]	Shape [*K*]
Uniform	(3.99 ± 0.01) · 10^4^	9.82 ± 0.03	45.67 ± 0.03	−3.29 ± 0.04
Gamma	(3.99 ± 0.01) · 10^4^	8.76 ± 0.03	44.92 ± 0.03	−2.81 ± 0.03

**Table 4:** First and second momenta and quantiles of histograms in Figure 7 and Figure 8.

	Mean	Std. dev.	Q1	Q2 (Median)	Q3	IQR
Uniform	37.64	6.30	34.20	38.95	42.81	8.62
Gamma	37.82	5.76	34.78	39.98	42.53	7.75

**Table 5:** Median values and IQR of the distribution of infected workers depending on the volume of patient zero’s room.

	Median (IQR)
Room 7	38.41 (8.38)
Room 9	38.49 (8.28)
Room 12	38.77 (7.92)
Room 16	39.06 (7.65)
Room 17a	39.47 (7.27)
Room 18	39.24 (7.55)
Room 21	39.12 (7.71)
Room 24	39.04 (7.48)
Room 27	38.96 (7.61)
Room 29	39.05 (7.52)
Room 32	39.52 (7.25)
Room 34	39.67 (7.31)
Room 39	37.52 (8.98)

**Table 6:** Fit results on the histogram of infected workers for the geriatric ward scenario with doubled ventilation (latency time = gamma distribution).

Normal. [*N*]	Scale [ω]	Location [ξ]	Shape [*K*]
(3.99 ± 0.01) · 10^4^	9.67 ± 0.04	41.57 ± 0.05	−2.81 ± 0.03

**Table 7:** Fit results on the histogram of infected workers for the geriatric ward scenario with workers wearing surgical masks (latency time = gamma distribution).

Normal. [*N*]	Mean [μ]	Sigma [σ]
(5.58 ± 0.02) · 10^3^	26.20 ± 0.02	7.14 ± 0.02

**Table 8:** Results of the sensitivity analysis.

Uniform distribution of latency times				
Parameter	O(q)	$O(q_{+})$	$O(q_{-})$	C[%]
*b*	37.71	37.74	38.54	2.12
Time diff.		37.83	37.68	0.40
**Gamma distribution of latency times**				
Parameter	O(q)	$O(q_{+})$	$O(q_{-})$	C[%]
δ	38.28	38.1	38.46	0.94
θ		37.86	38.43	1.49
Time diff.		38.23	38.06	0.45

## Data Availability

The data presented in this study are available on request from the corresponding author.

## Supplementary Materials

Details on source code can be found at: https://www.mdpi.com/article/10.3390/microorganisms12122401/s1.
