# Supplementary material for: An Easy-to-Use Tool to Predict SARS-CoV-2 Risk of Infection in Closed Settings: Validation with the Use of an Individual-Based Monte Carlo Simulation
Source: Microorganisms. 2024 Nov 22;12(12):2401. doi: 10.3390/microorganisms12122401 (PMC11678045; doi:10.3390/microorganisms12122401)
Supplement: Supplementary file 1 [file microorganisms-12-02401-s001.zip › microorganisms-3214040-supplementary.pdf]

Supplementary material

Santoro B., Larese Filon F. (corresponding), Milotti E.

1 Source code description

We developed the simulation code in C++, and used an object-oriented programming approach.

1.1 Classes

We defined three classes: one for the patients, one for the workers, and one for the room.

1.1.1 Patient class

We defined a *patient* class which collects the following variables:

- the room in which the patient is hospitalized;
- the infection status ι;
- the day on which the infection occurred;
- the latent time;
- the permanence time in the room.

Here is a snippet of the initialization code of this class:

```
#ifndef patient_h
#define patient_h
#include <stdio.h>
#include <stdlib.h>
#include <vector>
using namespace std;

class patient
{
public:
    patient(int cont);       // patient is initialized with a certain infection status
    ~patient();

    int contagiousness;     // infection status
    int room;               // hospitalization room
    int i_day;              // the day of the infection event
    double latent_time;     // patient's latent time
    double per_time;        // in hours
};
#endif
```

1.1.2 Worker class

Analogous to the *patient* class, we defined a *worker* class with the same parameters plus:

- a vector of the rooms visited each day;
- a parameter recording the room in which the worker was infected.

```
#ifndef worker_h
#define worker_h
#include <stdio.h>
#include <stdlib.h>
#include <vector>
using namespace std;

class worker
{
public:
    worker(int cont);  // worker is initialized with a certain infection status
    ~worker();

    int contagiousness;   // infection status
    vector<int> rooms;    // rooms visited during the day
    int infected_room;    // room where the infection occurred
};
#endif
```

1.1.3 Room class

The *room* class contains the following parameters:

- the volume;
- the number of infected individuals;
- the number of individuals who visited the room;
- the occupancy of the room.

Here is the snippet of the room class initialization:

```
#ifndef room_h
#define room_h
#include <stdio.h>
#include <stdlib.h>
#include <vector>
using namespace std;

class room
{
public:
    room(double volume, int n_occupants); // rooms initialized with their volume and occupancy
    ~room();

    double volume = 0;         // room volume
```

```
    int n_infected = 0;          // number of infected individuals in the room
    int n_visited = 0;           // number of individuals who entered the room
    int n_occupants = 0;         // number of hospitalized patients
};
#endif
```

1.2 Main code

1.2.1 Initialization

The simulation starts by setting the initial conditions:

- the number N_p of patients;
- the number N_w of workers;
- the environmental conditions such as ventilation, relative humidity, and personal protection equipment used by occupants;
- a *map* of rooms N_r with their volumes V_i $(i = 0, ..., N_r - 1)$.

Rooms are initialized using a map as shown below:

```
unordered_map<string,double> rooms = {{"0",V_0}, {"1",V_1}, {"2",V_2}, {"3",V_3}, {"
    4",V_4}, {"5",V_5}, {"6",V_6}, {"7",V_7}, {"8",V_8}, {"9",V_9}, {"10",V_10}, {"
    11",V_11}, {"12",V_12}};
```

Therefore three maps are created: one is filled with instances of the *patient* class, one of the *worker* class and one of *room* class. Patients and workers are initialised with infection status $\iota = 0$, meaning they are healthy and a latent time is randomly assigned to each occupant according to the distribution of latency period of choice. For our simulation scenario every room hosted two patients, therefore each room of the *map* is initialised with the corresponding volume and two occupants.

```
default_random_engine generator(seed);
gamma_distribution<double> distribution(2,3.3);
double t = distribution(generator);
patient pt = patient(0);
pt.latent_time = t;
```

Patients are randomly assigned to their room and patient zero is chosen; therefore his infection status is updated and latent time is set to zero.

1.2.2 Evolution of the epidemic

After initialization, a loop over the days of the outbreak starts. Each day a sub set A_w of workers is chosen and they are assigned to certain rooms extracted from the *map*.

```
unordered_map<int,worker> workers_allowed = allowed(wk);

/* rest of the code */

// function to extract a subset of the workers
unordered_map<int,worker> allowed(unordered_map<int,worker>& wk){

  unordered_map<int,worker> workers_allowed;
```

```
  while(workers_allowed.size() < 14)
  {
      int t = (rand()%wk.size());

      if(workers_allowed.find(t)!= workers_allowed.end()){

      }
      else{
        workers_allowed.insert({t,wk.at(t)});
      }

    }
  return workers_allowed;
}
```

The code loops over the number of admitted workers and for each room they visit the infection probability is computed as described in the Method section of the main text. At each visit, the number of visitors in the room is updated and if the infection occur the number of infected individuals is updated as well. If one of the occupants is infected is infection status ι is set to one and the *i_day* is set to the current day, while is contagiousness status is updated one day before the end of the latent time, see the main text. At the end of each simulation run the numbers of infected workers and infected patients and other information useful for the analysis, like the assigned latent times, are stored in a structured file.
